# Quantifying and Understanding Well-to-Well Contamination in Microbiome Research

**DOI:** 10.1128/mSystems.00186-19

**Published:** 2019-06-25

**Authors:** Jeremiah J. Minich, Jon G. Sanders, Amnon Amir, Greg Humphrey, Jack A. Gilbert, Rob Knight

**Affiliations:** a Marine Biology Research Division, Scripps Institution of Oceanography, University of California, San Diego, La Jolla, California, USA; b Department of Pediatrics, University of California, San Diego, La Jolla, California, USA; c Department of Ecology and Evolution, University of Chicago, Chicago, Illinois, USA; d Department of Surgery, University of Chicago, Chicago, Illinois, USA; e Center for Microbiome Innovation, Jacobs School of Engineering, University of California, San Diego, La Jolla, California, USA; f Department of Computer Science and Engineering, University of California, San Diego, La Jolla, California, USA; g Department of Bioengineering, University of California, San Diego, La Jolla, California, USA; University of Trento

**Keywords:** 16S rRNA gene, automation, built environment, contamination, genomics, low biomass, metagenomics, microbiome, microbiota, study design

## Abstract

Microbial sequences inferred as belonging to one sample may not have originated from that sample. Such contamination may arise from laboratory or reagent sources or from physical exchange between samples. This study seeks to rigorously assess the behavior of this often-neglected between-sample contamination. Using unique bacteria, each assigned a particular well in a plate, we assess the frequency at which sequences from each source appear in other wells. We evaluate the effects of different DNA extraction methods performed in two laboratories using a consistent plate layout, including blanks and low-biomass and high-biomass samples. Well-to-well contamination occurred primarily during DNA extraction and, to a lesser extent, in library preparation, while barcode leakage was negligible. Laboratories differed in the levels of contamination. Extraction methods differed in their occurrences and levels of well-to-well contamination, with plate methods having more well-to-well contamination and single-tube methods having higher levels of background contaminants. Well-to-well contamination occurred primarily in neighboring samples, with rare events up to 10 wells apart. This effect was greatest in samples with lower biomass and negatively impacted metrics of alpha and beta diversity. Our work emphasizes that sample contamination is a combination of cross talk from nearby wells and background contaminants. To reduce well-to-well effects, samples should be randomized across plates, samples of similar biomasses should be processed together, and manual single-tube extractions or hybrid plate-based cleanups should be employed. Researchers should avoid simplistic removals of taxa or operational taxonomic units (OTUs) appearing in negative controls, as many will be microbes from other samples rather than reagent contaminants.

**IMPORTANCE** Microbiome research has uncovered magnificent biological and chemical stories across nearly all areas of life science, at times creating controversy when findings reveal fantastic descriptions of microbes living and even thriving in what were once thought to be sterile environments. Scientists have refuted many of these claims because of contamination, which has led to robust requirements, including the use of controls, for validating accurate portrayals of microbial communities. In this study, we describe a previously undocumented form of contamination, well-to-well contamination, and show that this sort of contamination primarily occurs during DNA extraction rather than PCR, is highest with plate-based methods compared to single-tube extraction, and occurs at a higher frequency in low-biomass samples. This finding has profound importance in the field, as many current techniques to “decontaminate” a data set simply rely on an assumption that microbial reads found in blanks are contaminants from “outside,” namely, the reagents or consumables.

## Abstract (precis)

Microbiome research has uncovered magnificent biological and chemical stories across nearly all areas of life science, at times creating controversy when findings reveal fantastic descriptions of microbes living and even thriving in what were once thought to be sterile environments. Scientists have refuted many of these claims because of contamination, which has led to robust requirements, including the use of controls, for validating accurate portrayals of microbial communities. In this study, we describe a previously undocumented form of contamination, well-to-well contamination, and show that this sort of contamination primarily occurs during DNA extraction rather than PCR, is highest with plate-based methods compared to single-tube extraction, and occurs at a higher frequency in low-biomass samples. This finding has profound importance in the field, as many current techniques to “decontaminate” a data set simply rely on an assumption that microbial reads found in blanks are contaminants from “outside,” namely, the reagents or consumables.

## INTRODUCTION

Massively high-throughput sequencing has enabled fundamental changes to the study of microbial ecology. Increased throughput and sequencing depth have empowered researchers to utilize multiplexing to increase sample sizes to thousands per study (1–6). However, new ways of knowing require new understanding of potential flaws and confounding factors. Many studies have addressed computational and statistical challenges associated with analyzing 16S rRNA gene sequence data, including the impacts of sequence similarity clustering (7), diversity estimation (7), and data compositionality (8), to name just a few. There has also been substantial effort to reduce confounding experimental effects via standardization of microbiome sample processing methods, including sample collection, preservation (9), DNA extraction (10–12), library preparation (6, 13–17), and sequencing (5). Together, these approaches have facilitated large-scale meta-analyses such as the Earth Microbiome Project (EMP) (http://earthmicrobiome.org/) (2). Despite these efforts, a significant amount of experimental noise remains in any given microbiome study.

Contamination, or the observation of sequence reads in a sample coming from microbes that were not originally part of that sample, remains one of the most pernicious types of experimental noise. Microbial rRNA gene copies can be found even in “sterile” reagents, leading to the presence of a background signal derived from DNA extraction kits (18), PCR master mix (19), and other consumables (20). It is now widely understood that such contaminants must be considered in microbiome analyses, especially when dealing with low-biomass samples where contaminant rRNA gene copies make up a larger fraction of the community (7, 21–25). Various engineering strategies have been proposed and are utilized to minimize contamination, including physical separation of rooms used for DNA extractions and PCR, wearing additional personal protective equipment (PPE) (26) to cover skin to prevent technician-induced contaminants, UV sterilization of plastic consumables or reagents, or ethidium oxide treatment of consumables (20).

Beyond physically limiting contamination, positive and negative controls are increasingly being used to assess and quantify contamination in a study, allowing for the potential of contaminant removal *in silico* (27). Methods such as Katharoseq (11) utilize the ratio of read counts and composition of positive and negative controls to determine criteria for sample inclusion. Others have emphasized the importance of including negative controls to understand background contamination (28). Based on the idea that contaminants are primarily derived from external sources, some have proposed the strategy of simply identifying this “contaminome” profile and then removing them from the data set (29). This, however, fails to contend with the potential that contaminants may arise from other samples within a study itself. Such between-sample contamination has been observed as a product of “barcode swapping” between samples as a by-product of Illumina exclusion amplification sequencing reactions and has also been suggested to arise from improper assignment of barcodes to neighboring clusters in image processing (30). Anecdotally, we have also observed instances that appear to arise from physical cross-contamination of samples. Since most DNA extractions and PCRs are performed on multiple samples at once, oftentimes in a 96-well format, we reasoned that it would be important to take into consideration that nearby samples could in fact contribute to contamination of negative controls.

To evaluate this hidden factor of contamination, we designed an experiment to empirically characterize the frequency and nature of well-to-well contamination using different DNA extraction and sample handling protocols. By placing 16 unique bacterial “source” isolates at high biomass in individual wells across plates of alternating low-biomass “sink” bacteria and no-template blank wells, we were able to observe and quantify well-to-well transfer events under different scenarios, including automated plate-based extraction and manual tube-based extraction protocols. We further included libraries from an additional, unique, isolate that were extracted and amplified separately to account for potential instrument-based cross-contamination mechanisms such as barcode swapping or misassignment. To further validate results, we processed an additional two 96-well plates at another microbiome facility.

## RESULTS

**FIG 1:**
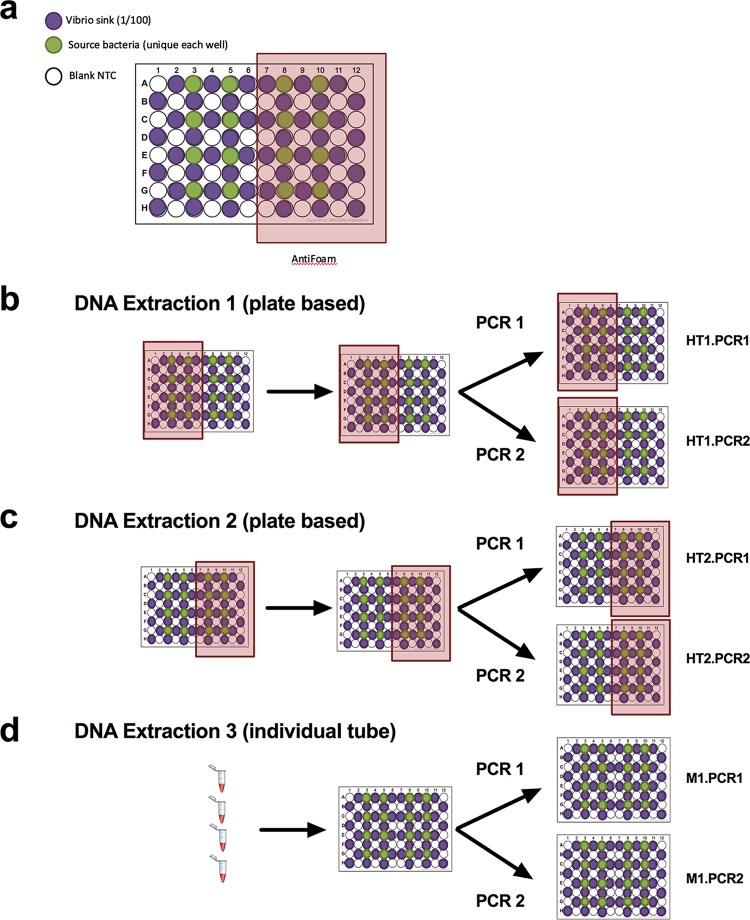
Plate design and experimental design. (a) NTC, sink, and source samples are distributed in a checkboard pattern across the plate. (b and c) Antifoam A is added to first half (b) and second half (c) of the 96-well plates processed with the robot in order to test whether antifoam A reduces foaming during bead beating and thereby well-to-well contamination. The manual samples did not receive antifoam A. Each unique DNA extraction plate is processed in duplicate PCR plates.

We designed a 96-well plate layout containing 16 unique source bacteria (∼10,000,000 cells per well, corresponding to 10^8^ cells ml^−1^), 24 sink wells (containing Aliivibrio fischeri at ∼100,000 cells per well [10^6^ cells ml^−1^]), and 48 blank wells (Fig. 1a). At the University of California, San Diego (UCSD), a total of three replicate sample plates were DNA extracted, two using the Epmotion5075 system with magnetic bead cleanups on Kingfisher robots (plate 1 and plate 2) and one manually with column cleanups (tube). All three extraction plates were then processed, each with two unique PCR amplifications (with each amplification consisting of pooled triplicate reactions, denoted PCRA and PCRB) (Fig. 1). In addition, 16 genomic DNA (gDNA) replicates of a *Clostridium* isolate were processed on its own 96-well plate and amplified in a separate PCR, to allow for detection of instrument-based barcode misassignment. A mock community comprised of all source isolates and the sink isolate was created and then serially diluted and processed as well to validate sample amplification. Details on the actual plate map patterns can be found in Fig. S1 in the supplemental material for all eight PCR plates. A total of 3,756,064 reads from 713 samples resulted in 6,305 unique 16S rRNA sequences (sub-OTUs [operational taxonomic units], or sOTUs). A summary table was generated to describe well-to-well and background contamination occurrences across the samples (Table S1). One of the 16 source microbes (Escherichia coli) was highly contaminated with background contaminants and did not produce the expected sequence results but was included in the analysis as we did not want to bias our results.

10.1128/mSystems.00186-19.1 FIG S1 Plate map descriptions of experimental design (96-well format). Sample wells are color coordinated by sample type (orange, blank/NTC; blue, sink/*A. fischeri*; purple, 1 of 16 source microbes). The top plate map was used for robot extractions. The second plate map was used for manual extractions. The third was used for mock community and sOTU taxon assignment. The fourth was used for barcode jumping testing. Download FIG S1, TIF file, 1.2 MB. Copyright © 2019 Minich et al. 2019 Minich et al. This content is distributed under the terms of the Creative Commons Attribution 4.0 International license.

10.1128/mSystems.00186-19.5 TABLE S1 Raw counts and summaries of contaminant profiles across samples, including metadata. Download Table S1, CSV file, 0.2 MB. Copyright © 2019 Minich et al. 2019 Minich et al. This content is distributed under the terms of the Creative Commons Attribution 4.0 International license.

**FIG 2:**
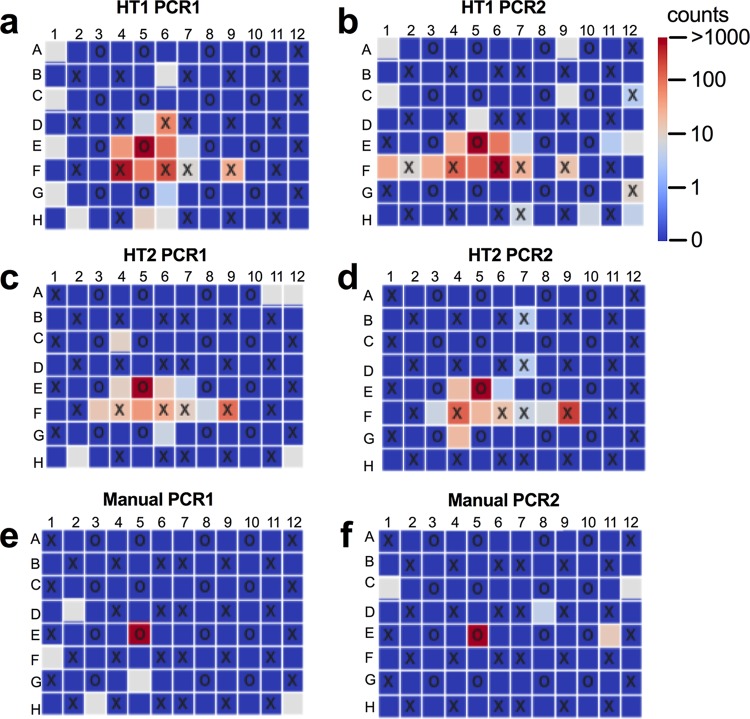
Example of plates with cross-contamination. Each panel depicts a 96-well plate, with source, sink, and blank wells denoted by “O,” “X,” and empty squares, respectively. Colors indicate the number of reads from a specific bacterium (*Psychrobacter* species, present in well E5). Panels a and b, c and d, and e and f correspond to two PCR replicates of robotic extractions 1 and 2 and manual extraction, respectively.

**FIG 3:**
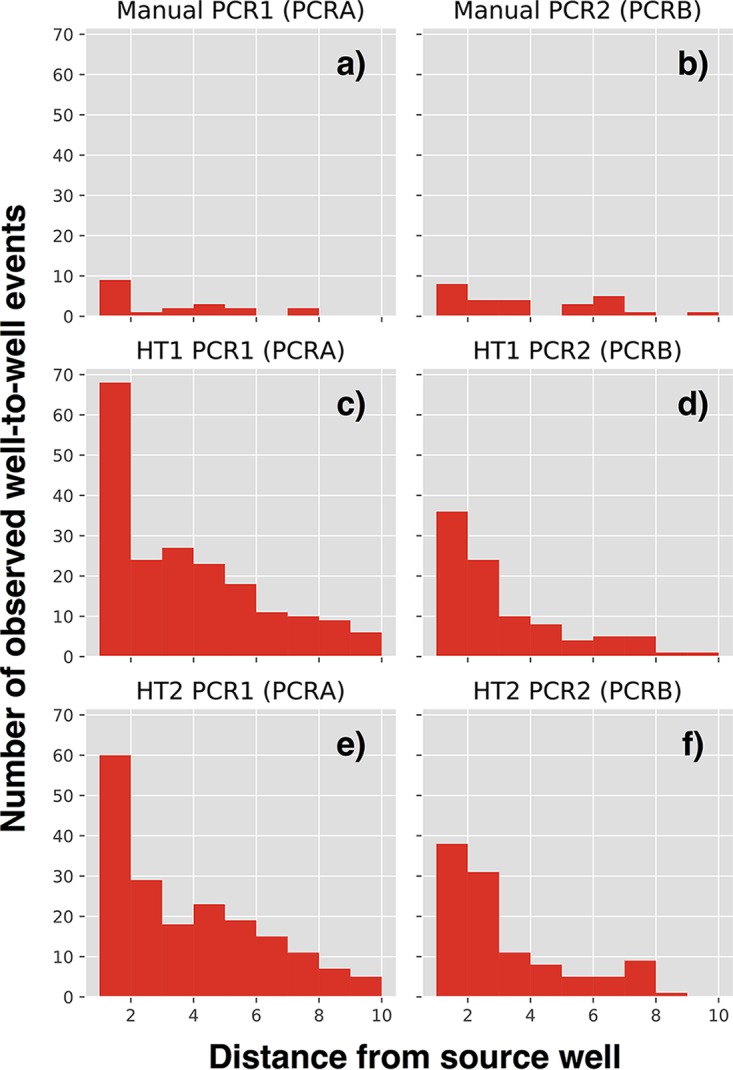
Distance-decay relationship of source samples contaminating surrounding samples. The distance (in units of wells) between “contaminant” observations of each sOTU and its source well was calculated. Histograms plot the number of inferred contamination events for each distance range for all 16 source microbes across the various DNA extraction plates and PCR replicate plate types from UCSD. Panels a and b, c and d, and e and f correspond to two PCR replicates of manual extraction and robotic extractions 1 and 2, respectively.

Well-to-well contamination events were analyzed by counting the fraction of reads from a given source well appearing in other source wells, low-biomass sink wells, or blanks. In our setup, well-to-well contamination occurred in all six PCR replicate plates in both laboratories. Based on the visualized plate patterns, the rate of well-to-well contamination was observed to be higher in plate extractions than in tube extractions and was more prominent in wells directly surrounding the source well, suggesting a physical mechanism for well-to-well contamination (Fig. 2). We quantified the distance by measuring contamination counts as a function of the Pythagorean distance from the source well and determined that the highest rates of contamination occurred in the immediately proximate wells for both plate and tube extractions but with a stronger distance-decay relationship for the plate than for the tube extractions (Fig. 3). The supplementation of antifoam A to wells during DNA extraction did not reduce well-to-well contamination (Fig. S2).

10.1128/mSystems.00186-19.2 FIG S2 Use of antifoam (antifoam = 1) does not reduce well-to-well contamination. Download FIG S2, TIF file, 0.4 MB. Copyright © 2019 Minich et al. 2019 Minich et al. This content is distributed under the terms of the Creative Commons Attribution 4.0 International license.

Another possible contributing source of intersample contamination is barcode leakage, i.e., reads originating from a given sample being identified as originating from a different sample due to read errors in the barcode. Such “barcode-hopping” behavior has been observed in laboratories using 8-bp barcodes in the Microbiome Quality Control project (31). In order to quantify the contribution of such events in our 12-bp barcode design, we designed another plate containing 16 replicate wells of a single *Clostridium* isolate. Since these samples were sequenced together with the extraction replicate plates, barcode leakage would be expected to result in *Clostridium* reads appearing in the extraction replicate plate samples. Barcode leakage was quantified by counting the number of reads originating from barcodes not present in the plate, and no such reads were observed, indicating that for the 12-bp Golay error-correcting barcodes sequenced under these conditions, this is a very rare event (<1 in 3.75e6 reads) and does not seem to be a factor contributing to intersample contamination using these parameters.

**FIG 4:**
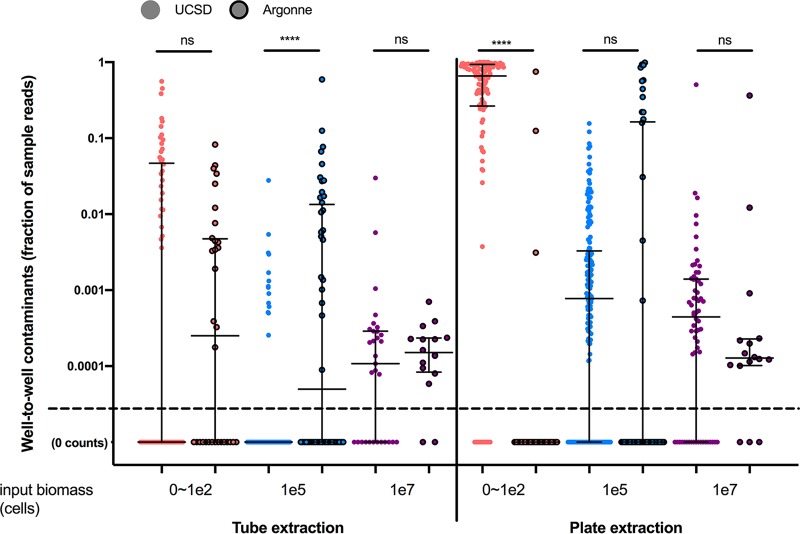
Summary statistics of sample fraction compositions of well-to-well contaminants compared across extraction types (blanks [pink], sink [blue], and source [purple]) and across extraction methods (tube versus plate). The *y* axis has a maximum value of 1 (corresponding to 100%). Sample types (NTC, sink, or source) were assigned an estimated input biomass of 0 to 100 cells, 1e5 cells, or 1e7 cells, respectively. For UCSD tube extractions, samples from both PCR replicate plates (PCRA and PCRB) were included. For UCSD robot plate extractions, samples from both PCR replicate plates and both DNA extraction plates were combined and organized by sample type. Argonne processed samples included one extraction plate and one PCR replicate plate. Samples processed at UCSD are indicated by circles with no outline, and samples processed at Argonne are indicated by circles with a dark border. All samples with zero well-to-well contamination occurrences are given a count of 0.00001 to enable visualization on the graph (labeled 0 counts). Medians and interquartile ranges are displayed in black lines over the data points. ****, *P* < 0.001; ns, not significant.

To further quantify the total effect of well-to-well contamination, we compared the proportions of reads within the microbial communities which were due to well-to-well contamination. For an initial overall experimental assessment, we compared UCSD to Argonne for each sample type, which had various biomasses (no-template controls [NTCs], ∼0 to 100 cells; sinks, ∼1e5 cells; sources, ∼1e7 cells), across the two major DNA extraction methods (manual single tubes versus robot plate) (Fig. 4). The frequency of well-to-well contamination was highest in low-biomass samples and generally higher in the robot plate-based extractions (Fig. 4; detailed summary statistics are provided for each DNA extraction plate and replicate PCR across all samples in Table S2). Contamination frequency and relative abundance were highest in plate 1 followed by plate 2 and lowest in the tube plate (Fig. S3). NTCs were composed of primarily background contaminants in the tube extractions for both PCR replicates (median fraction of well-to-well reads, 0). However, in some plate extraction NTCs, the majority of reads originated from well-to-well reads (median fractions of well-to-well reads of 0.78, 0.9, 0.44, and 0.77 for plate 1 PCRA and PCRB and plate 2 PCRA and PCRB, respectively). Sink wells were also partially contaminated with source microbes, particularly in the plate 1 replicate. The total occurrence (prevalence) of well-to-well contamination across the various sample types and extraction methods along with summarizing compositional effects of well-to-well contaminants on samples (mean, median, and maximum) are detailed in Table S2. For NTCs, 47.5% of blanks from tubes and 95.7% of blanks from plate extractions had well-to-well contamination. For low-biomass samples, 15.1% of sink wells from tubes and 67.4% of sink wells from plate extractions had well-to-well contamination (Table 1).

**TABLE 1:** Impact of contamination (well to well and background) on NTC, low-biomass, and high-biomass sample types^*a*^

Sample type (no. of samples)^*b*^	Location^*c*^	Extraction method^*d*^	Well to well						Background kit composition (%)		
			Mean prevalence (%)^*e*^	Richness		Composition (%)			Mean	Median	Max
				Avg no. of total unique reads	W2W%^*f*^	Mean	Median	Max			
NTC											
61	UCSD	m_tube	47.54	20	4.12	4.64	0.00	56.00	95.36	100.0	100.0
32	Argonne	m_tube	53.13	165	1.56	0.85	0.03	8.23	99.15	99.97	100.0
28	Argonne	m_plate	10.71	8	4.23	3.14	0.00	75.17	96.86	100.0	100.0
116	UCSD	Robot	95.69	15	27.79	63.79	74.78	100.0	36.21	25.22	100.0

Sink											
93	UCSD	m_tube	15.05	20	0.96	0.05	0.00	2.78	3.35	1.68	98.73
48	Argonne	m_tube	50.00	189	1.67	2.31	0.00	59.34	78.08	83.82	98.78
46	Argonne	m_plate	32.61	16	6.61	13.99	0.00	98.71	58.46	62.67	100.0
187	UCSD	Robot	67.38	15	12.70	0.70	0.08	15.61	0.93	0.25	40.51

Source											
31	UCSD	m_tube	61.29	18	6.51	0.13	0.01	2.99	8.30	0.29	100.0
16	Argonne	m_tube	87.50	21	13.78	0.02	0.02	0.07	11.54	0.41	99.98
16	Argonne	m_plate	81.25	17	16.79	2.37	0.01	36.40	13.13	0.32	99.99
64	UCSD	Robot	70.31	12	13.76	0.94	0.04	50.67	7.32	0.16	100.0

a Composition refers to the mean, median, or maximum frequency of sOTU contaminants that are due to well-to-well contamination or background kits.

b Refers to the total samples or well which had enough sequencing data for analysis.

c Location refers to the two laboratories which processed samples, either UCSD or Argonne.

d m_, manual (non-robotic-based extraction); Robot, robot-based DNA cleanup.

e Prevalence is calculated as the number of samples with any well-to-well contamination/total number of samples.

f W2W% is the percentage of total richness that is a result of well-to-well events, calculated as the number of unique well-to-well contaminants/total number of sOTUs (mean).

10.1128/mSystems.00186-19.3 FIG S3 Sources of contamination (well-to-well and background contaminants) across manual and robot extraction plates and PCR replicate plates (red, all background contaminant sOTUs; purple, all *A. fischeri* “sink” sOTUs”; black, all source sOTUs). Shown is a summary of the compositionality of NTCs (*n* = 48) versus sink microbes (*n* = 32) versus source microbes (*n* = 16) processed in two facilities across five DNA extraction plates: UCSD tube extraction (a), UCSD plate extraction 1 (b), UCSD plate extraction 2 (c), Argonne tube extraction (d), and Argonne plate extraction (e). UCSD DNA extractions were each processed twice and thus had two PCRs per plate (PCRA and PCRB). Download FIG S3, TIF file, 2.1 MB. Copyright © 2019 Minich et al. 2019 Minich et al. This content is distributed under the terms of the Creative Commons Attribution 4.0 International license.

10.1128/mSystems.00186-19.6 TABLE S2 Summary statistics on well-to-well contamination, including DNA extraction and PCR replicate plates from UCSD and Argonne. Download Table S2, CSV file, 0.00 MB. Copyright © 2019 Minich et al. 2019 Minich et al. This content is distributed under the terms of the Creative Commons Attribution 4.0 International license.

**FIG 5:**
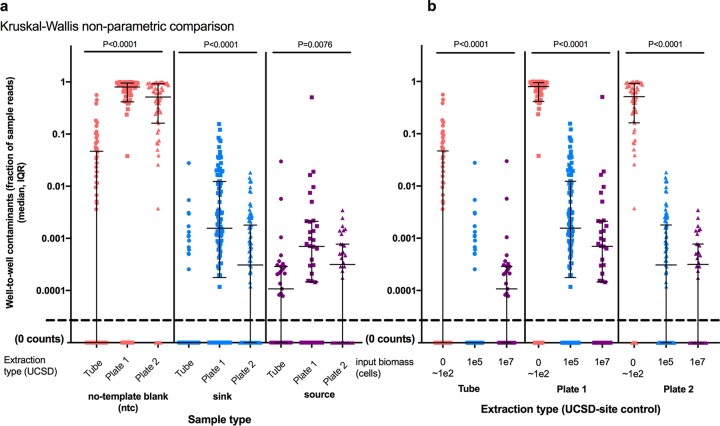
Well-to-well effect size. Shown are proportions of samples containing well-to-well contaminants organized by sample type (NTC, sink, and source) (a) and extraction method (b). The *y* axis has a maximum value of 1 (corresponding to 100%). Statistical analyses of data within bars were performed using Kruskal-Wallis nonparametric testing and indicate differences in contaminant fractions across extraction types (a) and among sample types (b). IQR, interquartile range.

To determine if DNA extraction method (tube versus plate) had an impact on well-to-well contamination, we compared relative abundances of well-to-well contaminants for NTC, sink, and source samples independently (Fig. 5a). The proportion of well-to-well contamination was affected by the extraction method and was generally higher in plate-based extractions than in manual single-tube extractions (*P* < 0.0001 by a Kruskal-Wallis test) (Fig. 5a). Furthermore, the proportion of well-to-well contamination was higher in samples with lower starting biomass (NTCs, 0 to 100 cells; sinks, approximately 100,000 cells) than in source wells, which had higher starting biomass (approximately 10,000,000 cells) while controlling for extraction method (Fig. 5b). Well-to-well contamination was greatest in samples with lower microbial biomass.

In order to validate these results in an independent laboratory, in addition to the samples processed at UCSD, we sent bacterial samples to be processed at an outside facility using manual single-tube extraction and plate extraction (although due to available facilities, both utilized a column cleanup step rather than magnetic beads). All results for replicate PCR plates and robot extraction replication were summarized for overall comparison purposes (Table 1). While controlling for site (UCSD only), the total fraction of reads from samples (mean, median, and maximum out of 100%) caused by well-to-well contamination was highest in NTCs, followed by sink and lastly source microbes for both the tube (NTC, 4.64%, 0%, and 56.0%, respectively; sink, 0.05%, 0.0%, and 2.78%; source, 0.13%, 0.01%, and 2.99%) and plate (NTC, 63.79%, 74.78%, and 100.0%; sink, 0.7%, 0.078%, and 15.61%; source, 0.94%, 0.04%, and 50.67%) extraction methods (Table 1 and Fig. 4). The NTCs of samples processed outside UCSD had well-to-well contamination consistent with those of the other tube methods, while the sink samples had higher rates of well-to-well contamination and overall background contamination than both tube- and plate-processed samples at UCSD (Table 1). For tube-extracted samples, there were significantly more well-to-well contamination events in sink samples at Argonne than at UCSD (*P* < 0.0001 by a Mann-Whitney test). For plate-extracted samples, there were more well-to-well events in NTCs at UCSD than at Argonne (*P* < 0.0001 by a Mann-Whitney test).

Since well-to-well contamination can introduce additional bacteria to samples, it has the potential to inflate alpha diversity and decrease resolution in beta diversity metrics, especially for binary metrics (such as number of observed species, Jaccard dissimilarity, or unweighted UniFrac distance). While all of our source and sink control samples should have had only one unique sOTU, richness was typically much higher than this due to contamination, including background kit contaminants along with well-to-well contaminants. We calculated the total richness per sample, which should have been 1, and determined the percentage of that richness which was due to well-to-well contamination. Both well-to-well contaminants and background kit contaminants contribute to this inflated richness. Controlling for site (UCSD only), we determined that well-to-well contamination inflated richness estimates for both tube- and plate-extracted samples. For sink and source samples, we expected a richness of 1 sOTU, but this can be inflated due to background contaminants, well-to-well contaminants, and the presence of multiple unique rRNA operons within a single genome. In our study, the sink samples processed with manual tube and plate methods had mean richness values of 20 and 15 sOTUs, with well-to-well contaminants making up 0.96% and 12.7% of these sOTUs, respectively. Source samples processed with manual tube and plate methods had mean richness values of 18 and 12 sOTUs, respectively, with well-to-well contaminants contributing averages of 6.51% and 13.76%, respectively. (Table 1). Thus, in plate-based extractions, which have smaller amounts of background contaminants, well-to-well contamination will contribute more to inflated alpha diversity.

We next assessed the impact of well-to-well contamination on beta diversity measurements of the communities. Specifically, for each unique DNA extraction plate, we performed pairwise comparisons of the PCR replicates for each of the three sample types, including NTCs and sink and source microbes for each unique well ID (metadata column name = well_ID). Because well-to-well contamination generally made up only a small proportion of the total reads of each sample, binary metrics (which tend to emphasize the impact of rare taxa) were more affected than abundance-weighted metrics (Fig. S4).

10.1128/mSystems.00186-19.4 FIG S4 Determining the origin of well-to-well contamination and its impact on distance metrics from 96 unique well_IDs across three DNA extraction plates and six PCR plates. (a) Summary comparison of the use of compositional (Bray-Curtis) or presence-absence (binary Jaccard) metrics to describe microbial communities from NTCs (red), sink microbes (lower biomass), or source microbes (higher biomass). (b) Determining the effects of well-to-well contamination from PCR processing only (PCR replicates) compared to the entire process of DNA extraction and PCR (DNA extraction replicates). The statistical tests were performed for dark colors only, while lightly shaded bars indicate the replicates for robot extraction plates. Download FIG S4, TIF file, 1.0 MB. Copyright © 2019 Minich et al. 2019 Minich et al. This content is distributed under the terms of the Creative Commons Attribution 4.0 International license.

To further elaborate on this observation and quantify where well-to-well contamination was coming from (PCR process only or DNA extraction), we compared replicate plates that were processed using the robot. This included two separate DNA extraction plates and then two PCR plates for each extraction plate. For each PCR replicate plate, 96 pairwise distances were computed and categorized by sample type for each of the two DNA extraction plates (Fig. S4b, light red shading). In addition, the pairwise distances from each of the 96 wells of the two replicate DNA extraction plates processed by the robots were also compared for the PCR replicate plate PCRA only. We found much less between-PCR than between-extraction variance, indicating that the effect of the combination of stochastic effects plus well-to-well contamination for DNA extraction is greater than for stochastic effects plus well-to-well contamination for PCR (Fig. S4b).

## DISCUSSION

Understanding experimental biases or noise in microbiome research is critical to drawing accurate inferences of the microbial world. Since microbes are found in nearly every ecosystem (2), it is extremely important to limit and ideally eliminate false positives in sample signatures. Contamination is a combination of background contaminants (DNA extraction kits, PCR master mixes, and enzymes), processing contaminants (equipment, air, and technicians), and plate contaminants (well-to-well contamination). In this study, we show that well-to-well contamination can play a major role in microbiome studies, especially when using plate-based DNA extraction methods and for samples with low starting biomass (when extracted alongside higher-biomass samples). This type of contamination is difficult to detect and relatively infrequently discussed but should be considered when designing and evaluating research. The majority of research to date has focused on identifying microbial contaminants in reagents and consumables (12, 18, 21) and subsequently using bioinformatics techniques to simply subtract out these contaminant taxa (22, 27, 32). Existing tools to remove contaminant taxa or OTUs (operational taxonomic units) from a data set largely focus on these background contaminants and do not yet consider the case of contamination from proximal wells (27). We show in this study that a large fraction of reads in blank (NTC) samples originate from neighboring wells. In this study, we observed that contamination between samples can account for a significant fraction of the overall observed diversity in a sample, especially for no-template control blanks that are physically adjacent to relatively high-biomass samples. Given this, the simple approach of removing any taxa found in blanks is likely to remove the most prominent “real” taxa in a data set. More-sophisticated methods using additional information (such as the “decontam” package [27]) should be developed and updated to take into account contamination from nearby samples in the face of well-to-well contamination, even for addressing the problem of reagent contaminants.

Identifying and removing well-to-well contamination *in silico* are challenging, as contamination events between wells are largely independent and thus cannot be statistically identified and removed across a study in the same way that reagent contaminants are. However, several observations from this experiment should help researchers in planning experiments to minimize its effects.

First, plate-based DNA extractions are much more susceptible to well-to-well contamination than the more labor-intensive and human-reliant tube-based extractions. Although, in this experiment, we were not able to identify at precisely which step contamination occurred in the plate-based extraction, for critical experiments, automated plate-based extractions should be carefully reconsidered or optimized to reduce contamination. For low-biomass samples that require magnetic bead-based cleanups (11), we recommend a hybrid protocol whereby lysis occurs in single tubes to reduce well-to-well contamination followed by magnetic bead cleanup in plates, specifically with the KingFisher robot (catalog number 5400630 or 5400640; Thermo Fisher).

Second, well-to-well contamination was greatest in wells immediately adjacent to the source. Although the strength of this relationship was substantially stronger in plate-based extractions (Fig. 3), consistent with the bulk of local contamination happening at the plate extraction stage rather than in segments of the protocol that were plate structured in both protocols, there was still some bias toward neighboring-well contamination in the tube-based replicates. Thus, sample location on plates should be explicitly considered in experimental design. When plating samples for extraction, it is important to block and/or randomize treatments across 96-well plates. This will better ensure that well-to-well contamination adds only noise and not bias to experimental designs. If, for instance, all “treatment” samples are plated together on one half of a plate and “control” samples are plated on the other, well-to-well events between nearby wells will tend to artificially increase community similarity metrics within treatment and control groups, increasing the likelihood of detecting a false-positive signal.

Third, well-to-well contamination has the greatest impact on low-biomass samples, especially when they are processed adjacent to high-biomass samples that can act as sources. Because of this, it is important to have an awareness of the absolute concentration of microbial cells in samples and to ensure that only samples of similar biomasses are processed together. Although low-biomass samples are most impacted as a fraction of total reads, high-biomass samples such as fecal or soil samples may still show impacts of well-to-well contamination among lower-abundance taxa.

Finally, when analyzing data sets, it is important to be aware that different methods will have different sensitivities to well-to-well contamination. For example, alpha diversity estimates can be highly inflated by well-to-well contamination in samples with low starting diversity. For beta diversity estimates, binary metrics such as Jaccard dissimilarity or unweighted UniFrac distance are more likely to be affected than abundance-weighted metrics. Furthermore, different sites and sequencing runs may also contribute to varied well-to-well contamination frequencies. Other experimental approaches to reduce the impacts of well-to-well contamination bear further investigation. These might include the use of higher-fidelity liquid handling approaches (33) or broader adoption of unique per-sample positive-control spike-ins to allow the direct observation and statistical disambiguation of cross-contamination (34). Methods which rely on identifying and subtracting putative contaminants from data sets need to be used with extreme caution, particularly if the identified sequence variants are present in primary samples.

Understanding experimental noise is extremely important for improving and guiding microbiome research best practices (23, 24). Specifically, addressing “hot” negative controls is one of the great challenges to genomics-based research. Since well-to-well contamination is an important component of this, we emphasize that for any given experiment, it is critical to identify any kit-specific background contaminants in a lot to best accurately remove contaminant taxa. While we have good power to estimate the frequency of well-to-well contamination in our assays, extrapolating the frequency of well-to-well contamination in assays from other laboratories and methods is still a challenge. This suggests that while we can generalize well-to-well contamination as being a widespread problem, we cannot generalize the quantities or specifics. Furthermore, this argues for other laboratories spending the effort to perform similar in-house tests to evaluate their own pipelines. To identify these background contaminants, we recommend using a variety of positive-control titrations at both the DNA extraction stage and the PCR stage (11). Companies that manufacture high-throughput DNA extraction assays will need to invest in research and development to reduce well-to-well contamination. Finally, measuring and accounting for well-to-well contamination identification and reduction will be critical for diagnostic research going forward (35–40).

### Conclusions.

Contamination is a serious impediment to reproducibility in any genomics study, particularly microbiome research. As emerging diagnostic tests for environmental health and human health become more mainstream, it will be crucial for these tests to address variability in microbiome signals due to well-to-well contamination. Our study identified and quantified a previously underreported and underappreciated source of contamination in microbiome studies. We show that the intensity of well-to-well contamination varies per extraction method, with plate-based methods and lower-biomass samples having higher rates of contamination. Our findings demonstrate the importance for the community to accept standards to best monitor and quantify these sources of noise in a given study.

## MATERIALS AND METHODS

### Sample collection and processing.

A total of 17 bacterial isolates, including isolates of *Brevibacterium* sp., Corynebacterium stationis, *Brachybacterium* sp., *Arthrobacter* sp., Propionibacterium acnes, *Bacillus* sp., Staphylococcus equorum, Staphylococcus succinus, Streptococcus anginosus, Desulfovibrio sulfodismutans, *Serratia* sp., *Halomonas* sp., *Psychrobacter* sp., Pseudomonas fragi, Vibrio rumoiensis, Escherichia coli, and Aliivibrio fischeri, were collected and stored in a phosphate-buffered saline (PBS) solution. The optical density at 600 nm (OD_600_) was measured for all isolates, and the corresponding cell density was estimated. Sixteen of these microbes (all except A. fischeri) were diluted to a final density of 1e8 cells per ml in a single 50-ml conical vial and designated “source” organisms. The *A. fischeri* isolate was diluted to 1e6 cells per ml, designated the “sink” microbe, and stored in a single 50-ml conical vial. Both source and sink microbes were stored in a −80°C freezer until aliquots were made for extractions. In addition, a mock community was created using these isolates by combining equal volumes of all samples, which also served as a reference for accounting for processing biases. An additional isolate of *Clostridium* sp. was measured and aliquoted into 16 different 2-ml tubes to be used for barcode testing. For DNA extraction at UCSD, 100 μl of source and sink samples was aliquoted into 2 96-well DNA extraction robot plates and 96 2-ml bead-beating extraction tubes, as indicated in the diagram (Fig. 1a; see also Fig. S1 in the supplemental material). Following the Earth Microbiome Project protocol (2), the Qiagen PowerMag kit (catalog number 27500-4-EP) was used for robot extractions, while the Qiagen DNeasy PowerSoil kit (catalog number 12888-100) was used for “manual, single-tube” extractions. To test the effect of antifoam on reducing well-to-well contamination, we added 2 μl of an antifoam A concentrate (catalog number A5633-25G; Sigma-Aldrich) to half of each of the robot plates (Fig. 1b and c). In addition to processing samples at UCSD, an additional 192 samples were plated (96 unique samples in duplicate) in a 96-well plate and 96 individual 2-ml bead-beating tubes and sent to Argonne National Laboratory in the same plate map scheme. The manual tube samples were processed using the Qiagen DNeasy PowerSoil kit (catalog number 12888-100), while the manual plate samples were processed using the Qiagen DNeasy PowerSoil HTP 96 kit (catalog number 12955-4).

### Amplicon sequencing.

To distinguish between well-to-well contamination derived from DNA extraction and that derived from the PCR setup, each UCSD-processed DNA extraction plate (2 robot plates and 1 manual plate) was subjected to two separate triplicate PCRs, labeled PCRA or PCRB (Fig. 1b to d). The mock community dilution plate and barcode testing plate were processed with a single triplicate PCR each. The EMP 16S rRNA V4 primers 515f and 806rB were used to amplify the samples. Equal concentrations of amplicons from each sample from all 8 plates were pooled and sequenced using the MiSeq platform (5, 13, 14). The 192 samples DNA extracted at Argonne were processed using the same EMP primers and method but on a separate MiSeq run. Amplicon data were uploaded to Qiita (41) and processed with Qiime 1.9.1 (42). Exact sequence tags from the first read (150 bp) were generated using the Deblur pipeline with default parameters as described previously (43).

### Statistical analysis.

Sequences processed with Deblur were positively filtered against the reference database as part of the default workflow in Deblur. In addition, singleton sequences were omitted from the data set. The data set was not rarified in order to best quantify well-to-well contamination for all samples processed. The sequence tags were identified for all of the positive controls used in this study and are included in Table S2 in the supplemental material. Sequences which did not have a 100% match to those original controls were considered “background contaminants,” whereas the *A. fischeri* sequences were deemed “sink microbes,” and the 16 unique isolates were collectively deemed “source microbes.” For each of the 16 source microbes, 1 sink microbe, and 1 barcode leakage microbe, a custom script was used to generate 96-well plate maps to visualize well-to-well contamination. The distances of microbial dispersal “jumping” were then calculated for each individual isolate using a custom script. Summary statistics of read counts, richness, and contamination metrics are summarized in Table S2 in the supplemental material. To determine if the rate of well-to-well contamination was higher in robot than in manual extractions, the compositions of well-to-well contaminants were compared within no-template control (NTC), sink, and source samples independently, using the Kruskal-Wallis test. Furthermore, to determine if well-to-well contamination was associated or more frequent with lower-biomass samples, well-to-well compositions were compared across the NTC, sink, and source samples within each extraction method independently using the Kruskal-Wallis test.

To determine the impact of well-to-well contamination on beta diversity microbiome analyses, we calculated both Bray-Curtis (44, 45) and Jaccard (46) distance metrics and compared them within categories. The three different extraction plates each had two separate PCR plates processed. The pairwise distances of unique well_IDs were calculated using both metrics for each of the two PCR plates belonging to each of the three DNA extraction plates. Sample types were grouped into NTC (or blank), sink, or source. Within each group, the distances were compared using the Mann-Whitney test. To calculate effects for the entire pipeline, which includes both PCR and DNA extraction, we combined the pairwise distances of the well_IDs for each of the three DNA extraction plates (robot 1, robot 2, and manual) and grouped them by sample type (NTC, sink, or source). Again, we compared the total dissimilarities of Bray-Curtis versus Jaccard for each sample type using a Mann-Whitney test.

### Data availability.

All data have been made publicly available at the EBI database (accession number ERP115213) and through Qiita (Qiita accession number 10401 [https://qiita.ucsd.edu/study/description/10401]).
