# Supplementary figures and images for: Quantifying and Understanding Well-to-Well Contamination in Microbiome Research
Source: mSystems. 2019 Jun 25;4(4):e00186-19. doi: 10.1128/mSystems.00186-19 (PMC6593221; doi:10.1128/mSystems.00186-19)

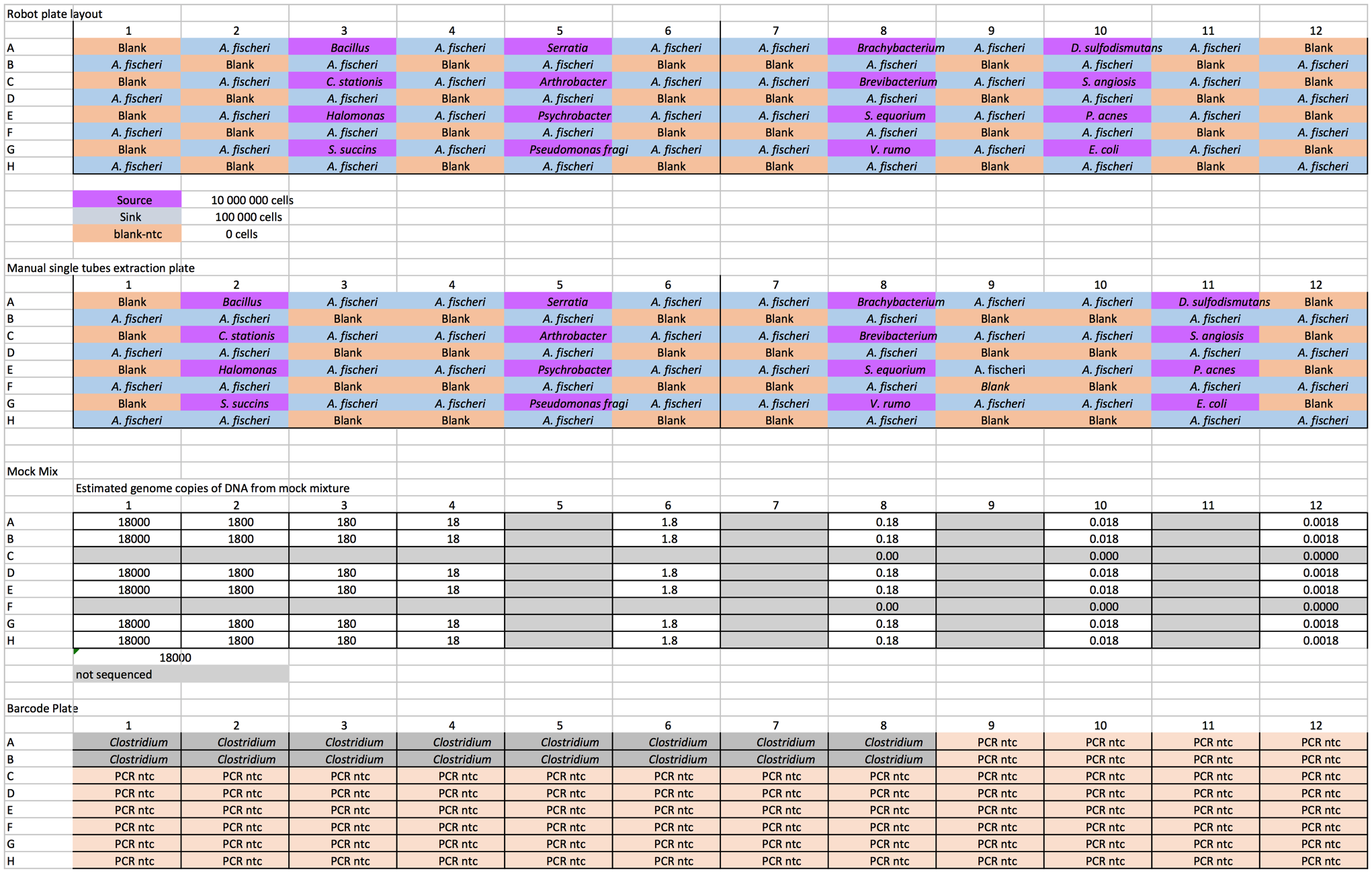

Supplement: FIG S1 [file mSystems.00186-19-sf001.tif]

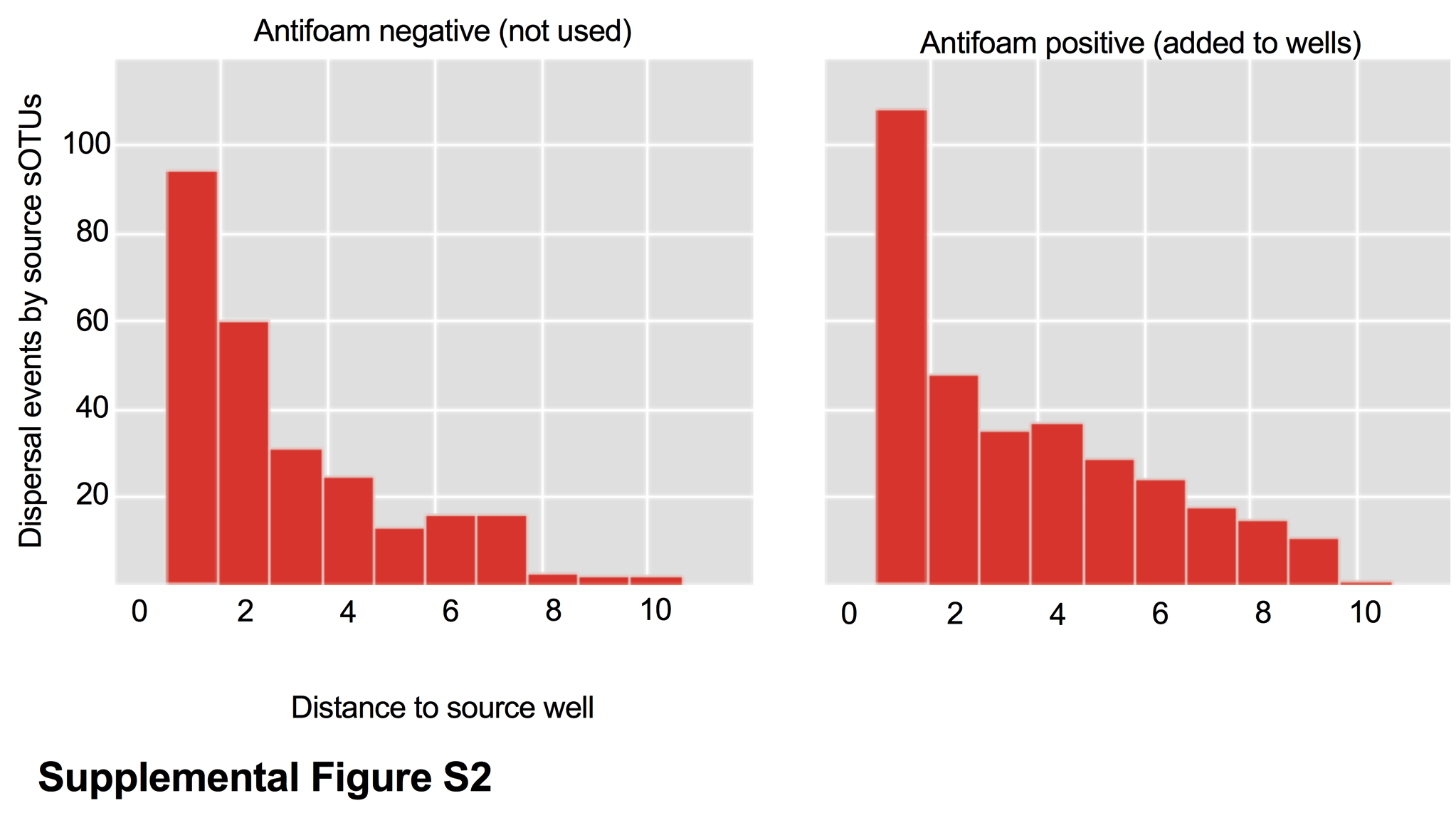

Supplement: FIG S2 [file mSystems.00186-19-sf002.tif]

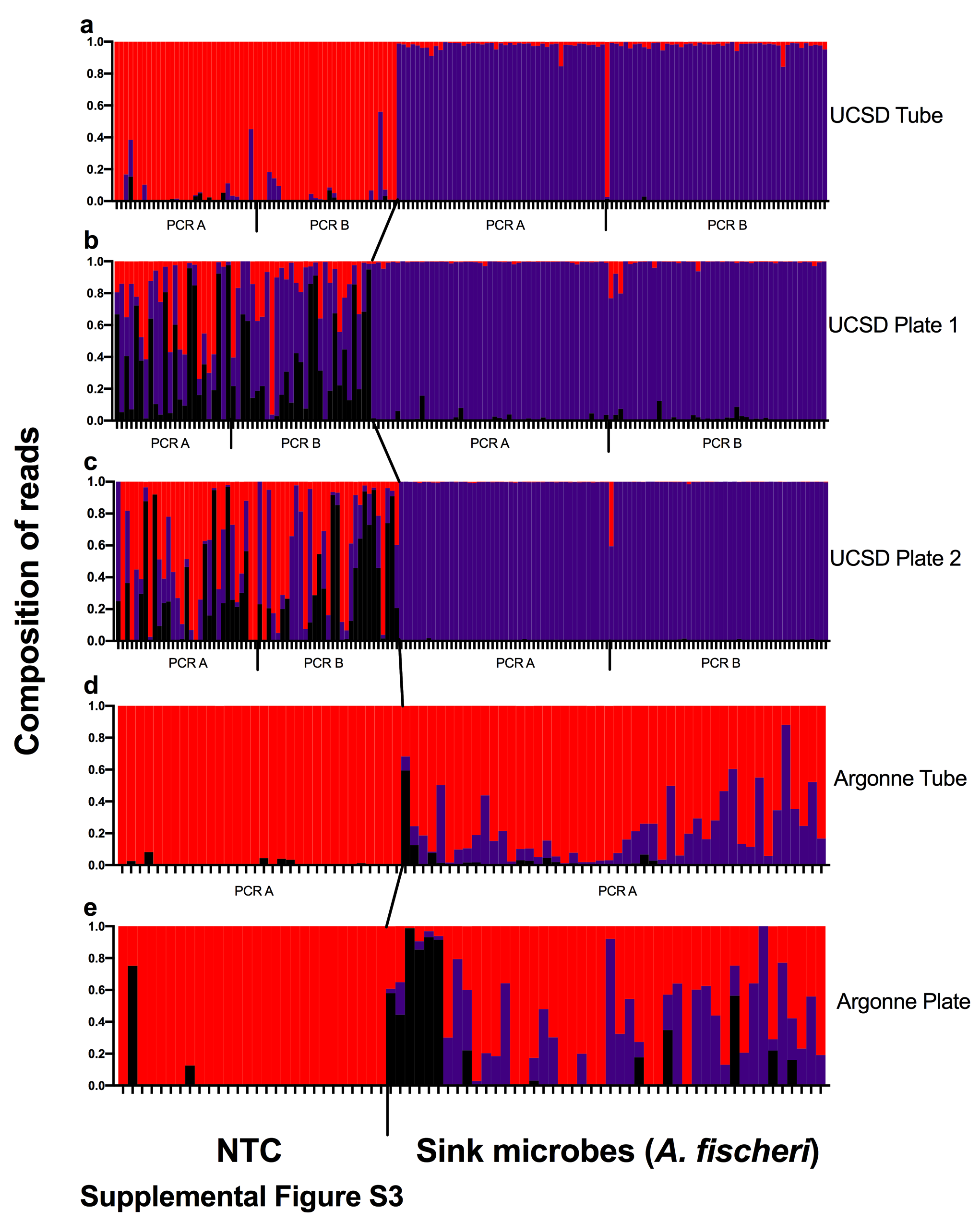

Supplement: FIG S3 [file mSystems.00186-19-sf003.tif]

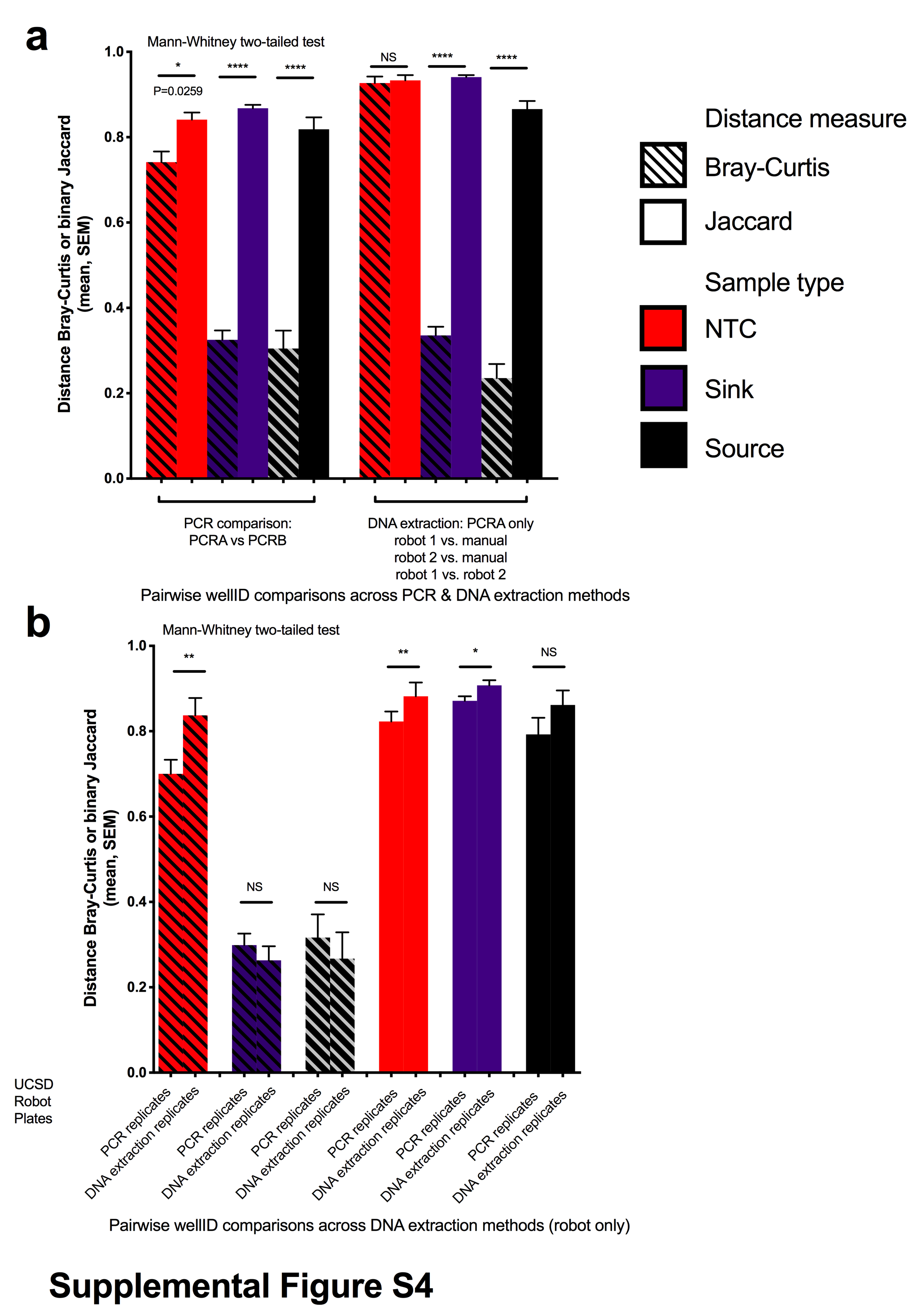

Supplement: FIG S4 [file mSystems.00186-19-sf004.tif]
